# Missed Opportunities for Retention in Pre-ART Care in Cape Town, South Africa

**DOI:** 10.1371/journal.pone.0096867

**Published:** 2014-05-07

**Authors:** Elizabeth du Toit, Cari van Schalkwyk, Rory Dunbar, Karen Jennings, Blia Yang, David Coetzee, Nulda Beyers

**Affiliations:** 1 Desmond Tutu Tuberculosis Centre, Department of Paediatrics and Child Health, Stellenbosch University, Parow, South Africa; 2 The South African Department of Science and Technology/National Research Foundation Centre of Excellence in Epidemiological Modelling and Analysis (SACEMA), Stellenbosch University, Stellenbosch, South Africa; 3 City of Cape Town Health Directorate, Cape Town, South Africa; 4 School of Public Health and Family Medicine, University of Cape Town, Cape Town, South Africa; Boston University, United States of America

## Abstract

**Background:**

Few studies have evaluated access to and retention in pre-ART care.

**Objectives:**

To evaluate the proportion of People Living With HIV (PLWH) in pre-ART and ART care and factors associated with retention in pre-ART and ART care from a community cohort.

**Methods:**

A cross sectional survey was conducted from February – April 2011. Self reported HIV positive, negative or participants of unknown status completed a questionnaire on their HIV testing history, access to pre-ART and retention in pre-ART and ART care.

**Results:**

872 randomly selected adults who reported being HIV positive in the ZAMSTAR 2010 prevalence survey were included and revisited. 579 (66%) reconfirmed their positive status and were included in this analysis. 380 (66%) had initiated ART with 357 of these (94%) retained in ART care. 199 (34%) had never initiated ART of whom 186 (93%) accessed pre-ART care, and 86 (43%) were retained in pre-ART care. In a univariable analysis none of the factors analysed were significantly associated with retention in care in the pre-ART group. Due to the high retention in ART care, factors associated with retention in ART care, were not analysed further.

**Conclusion:**

Retention in ART care was high; however it was low in pre-ART care. The opportunity exists, if care is better integrated, to engage with clients in primary health care facilities to bring them back to, and retain them in, pre-ART care.

## Introduction

South Africa had an estimated HIV incidence of 0.94% in adults in 2012 [1] with an estimated 6.1 million people living with HIV (PLWH) of which 2.2 million were on antiretroviral therapy (ART) [2]. There has been a strong drive to increase the numbers of people testing for HIV and HIV counselling and testing (HCT) occurs at all levels of the health care system, and within different health care programmes such as the Prevention of Mother to Child Transmission (PMTCT) programme and the Tuberculosis (TB) programme. It is also offered at community sites, through outreach drives and at the work place. This presents challenges to ensure that clients who undergo HCT, irrespective of where it is conducted, are linked to the appropriate follow up package of care, support and treatment [1]. More focus is given within programmes to PLWH who are on ART to ensure adherence to therapy than to retention in pre-ART care for those not yet qualifying for ART.

Studies in South Africa show that the majority of persons who initiate ART have low CD4 cell counts [3], [4]. A report from the IeDEA and ART cohort collaborations show that in South Africa in 2007 women initiated ART at a median CD4 count of 124 cells/mm^3^ and men at 111 cells/mm^3^ [5]. Recent changes in guidelines increasing CD4 eligibility to initiate ART may help to improve this. Poor access to, or poor retention in pre-ART care, may contribute to the low baseline CD4 at ART initiation, which is associated with high mortality [6].

The standard of HIV care (2013) in South Africa includes HCT, care prior to initiation on ART (pre-ART care) and initiation and maintenance on ART (ART care). The target is for 80% of men and women aged 15–49 years (30 million) to have been tested for HIV by 2016 [1]. CD4 cell count monitoring should occur at six monthly intervals in PLWH not yet eligible for ART and they should be retained in pre-ART care [1], [6], [7].

The majority of national ART programmes in sub Saharan Africa report the number of people initiated and retained on ART with few reporting on pre-ART retention [8]. Pre-ART studies looking at the proportion of PLWH who have had a CD4 count after HIV diagnosis have reported a range from 55–78% [9]–[11]. Studies reporting on PLWH who returned for a follow up CD4 count have ranged from 26–45% [12], [13].

A systematic review looking at retention in HIV care between testing and treatment in sub-Saharan Africa estimated that only a sixth to a third of patients who test positive for HIV are staged, and enroll and remain in pre-ART care until they are eligible for and have initiated ART [8]. There is little data on retention in pre-ART care and more research is required.

We evaluated the proportion of PLWH in pre-ART and ART care and factors associated with retention in pre-ART care from a community cohort.

## Methods

### Setting, population and study design

This study was conducted in the Western Cape Province of South Africa in eight communities with a high burden of HIV and TB in the greater Cape Town area. All communities are served by at least one public sector primary health care facility, which offers HCT, pre-ART and ART care. This survey was conducted in 2011 when the South African guidelines stipulated that PLWH with a CD4 count lower than 200 cells/mm^3^; or 350 cells/mm^3^ in patients with TB or who were pregnant; or in patients with WHO stage 4 disease irrespective of CD4 count, should commence ART [7]. The change to initiate ART at CD4 <350 cells/mm^3^ and for all with tuberculosis or pregnant or breastfeeding [14], was introduced after this survey was completed.

The sampling frame for this study comprised adults who were previously part of a TB and HIV prevalence survey in 2010 as part of the Zambia South Africa TB and AIDS Reduction (ZAMSTAR) study [15] and whose self reported HIV status was known from that survey. Randomly selected adults (≥18 years old) from each of the eight communities of the 2010 ZAMSTAR prevalence survey [16] who had disclosed that they were HIV positive and agreed to be contacted again, were revisited between February and April 2011. After written informed consent was obtained, they were asked to complete a questionnaire that focused on their HIV testing history, access to pre-ART and retention in pre-ART and ART care, as well as the last time they had attended a public sector health care facility. In order to decrease stigmatisation that may have resulted from visiting only the homes of self disclosed HIV positive adults, in each community a small number of randomly selected self disclosed HIV negative adults or adults who did not know their HIV status were also revisited and asked to complete the same questionnaire. However, only those who self reported that they were HIV positive at the revisit were included in this analysis.

### Data collection and management

Lists of names of the randomly selected adults were generated and unique barcodes printed for each selected adult. Trained research assistants received the barcodes and management forms and a separate list with the names and addresses of the selected adults. The research assistants were unaware of the participant's HIV status and visited each participant in his/her home where they barcoded the forms, obtained written informed consent, interviewed the participant in their home language and completed the questionnaire. The questionnaire data were recorded using the scanned barcodes (no personal identifiers) on an electronic personal data assistant (PDA) and downloaded on a daily basis onto a server at the Desmond Tutu TB Centre (DTTC). Only the data manager could link the unique barcode to the participant's name from the informed consent form. Data from the questionnaires were merged by the data manager to data (age, sex, race, years lived in the area, and history of previous TB treatment) from the ZAMSTAR 2010 prevalence survey.

### Definitions

ART group: PLWH who self reported that they had been initiated onto ART.

Retention in ART care: People in the ART group who self reported that they had collected ART medication within the last three months of being interviewed.

pre-ART group: PLWH who self reported that they had never initiated ART.

Accessed pre-ART care: PLWH who self reported that they had had at least one CD4 count.

Retention in pre-ART care: People in the pre-ART group who self reported that they had had a CD4 count within 6 months of being interviewed.

### Ethics Statement

All participants gave written informed consent during the ZAMSTAR 2010 Prevalence survey to be visited again for follow up studies. All participants gave written informed consent for this Access and Retention in Care Study. The study was approved by the Stellenbosch University Committee for Human Research, the Ethics Advisory Group of the International Union against TB and Lung disease (The Union) and the Human Research Ethics Committee at the Health Sciences Faculty of the University of Cape Town.

### Statistical analysis

Stata version 12 (Stata Corp.LP, College Station, TX, United States of America) was used for all analyses. Standardisation was used to account for the sex ratio of HIV prevalence in the Western Cape according to the ASSA model [17]. Logistic regression with robust standard errors to control parameter estimates for clustering at a community level was used to explore factors associated with retention in HIV care in the pre-ART group. The impact of the following factors on the likelihood of retention in pre-ART care was explored using a univariable regression: sex; age; employment status; highest education level; years living in the same area; history of previous TB treatment; mode of transport and cost to reach the clinic at their last visit, overall waiting time at the clinic; and knowing someone close to them who either had HIV, was on ART or had died from HIV.

## Results

**Figure 1:**
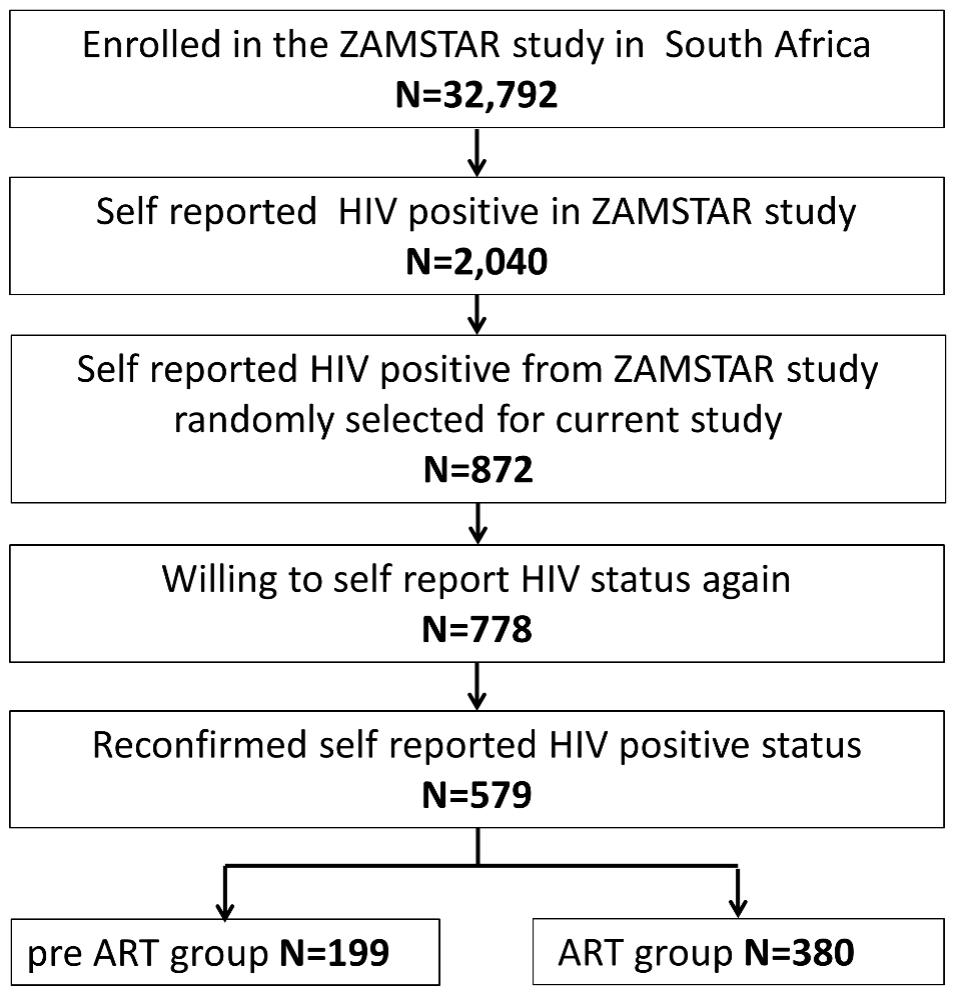
Participant Flow Chart.

872 randomly selected adults who reported that they were HIV positive in the ZAMSTAR 2010 prevalence survey were included and revisited (Figure 1). No statistical significant differences in age, sex, employment or education were found between the group that was randomly selected for this study and the group that was not selected.

778 (89%) were willing to disclose their HIV status at the follow up interview. 579 (66%) reconfirmed their positive status and were included in this analysis. This group had significantly more females than the 293 (34%) not included (87% vs 72%, p<0.001) and significantly more unemployed (66% vs 42%, p<0.001).

Of the 579 participants included in this analysis 501 (87%) were female (Table 1). The mean age of all participants was 36 years with 445 (77%) between 25 and 44 years of age. 452 (78%) had lived in the community for more than 5 years. 174 (30%) had only ever tested once for HIV, 237 (41%) had tested twice, and 162 (28%) had tested 3 or more times.

**Table 1:** Demographic and HIV testing characteristics of study participants.

Study Participants (n = 579)	Subcategory	N (%)
**Sex**	Female	501 (87)
	Male	78 (13)
**Age categories in years**		
	18–24	42 (7)
	25–34	224 (39)
	35–44	221 (38)
	>45	92 (16)
**Employment status**		
	Unemployed	380 (66)
	Employed either part time or full time	199 (34)
**Years living in the community**		
	<5 years	120 (21)
	>5 years	452 (78)
	Missing data	7 (1)
**Number of times participants had tested for HIV**		
	1	174 (30)
	2	237 (41)
	3	118 (20)
	4	29 (5)
	>4	15 (3)
	Unknown or missing data	6 (1)

380 of the 579 participants (66%) were in the ART group of whom 357 (94%) were retained in ART care (Table 2). Of the 357 retained in ART care 313 (88%) were female, the mean age was 36.2 years, 281 (79%) had secondary education, and 243 (68%) were unemployed.

**Table 2:** Retention in ART and pre-ART care.

Study Participants (n = 579)	Subcategory	N (%)
**ART group**		**380 (66%)**
	Collected ART medication within the last three months of being interviewed	357 (94%)
	Did not collect ART medication within the last three months of being interviewed	23 (6%)
**pre-ART group**		**199 (34%)**
	Had a CD4 count within the last 6 months of being interviewed	86 (43%)
	Did not have a CD4 count within the last 6 months of being interviewed	113 (57%)
**Access to pre-ART care**		**199**
	Accessing pre-ART care (reported to have had at least one CD4 count)	186 (93%)
	Did not access pre-ART care	13 (7%)

Due to the high retention in ART care, factors associated with retention in ART care, were not analysed further

199 (34%) participants never initiated ART, of whom 186 (93%) accessed pre-ART care as evidenced by self reporting that they had had at least one CD4 count in the past. 141 (76%) knew the value of their last CD4 count, the mean was 548 cells/mm^3^ and the range from 101–2000 cells/mm^3^. 124 (67%) reported that their first CD4 count had been taken on the same day that they had tested positive for HIV. Two (1%) did not know if they had ever had a CD4 count. Of those having had a CD4 count, 57 (31%) had only ever had one. Of the 186 people in the pre-ART group who had accessed pre-ART care, 86 (43%) were retained in pre-ART care (Table 2). Of the 86 retained in pre-ART care 80 (93%) were female, the mean age was 33.2 years, 75 (87%) had secondary education, and 51 (59%) were unemployed.

Even though 113 in the pre-ART group were not retained in pre-ART care 78 of these (69%) had accessed general health care by attending a health care facility for any medical reason within the last three months, but did not have a CD4 count done at that attendance.

In the univariable analysis (Table 3) women were more likely to be retained in pre-ART care (OR = 3.0), but this association was not statistically significant, possibly due to the small sample size of the group under consideration. None of the other factors analysed were significantly associated with retention in care in the pre-ART group.

**Table 3:** Univariate analysis of factors that might be associated with retention in pre-ART care.

			UNIVARIATE			
	pre-ART group	Retention in pre-ART care				
	N (%)	n (% of N)	OR	95% CI		p-value
**Total**	199	86 (43%)				
**Sex**						
**Male**	27 (14%)	6 (22%)	1.0			
**Female**	172 (86%)	80 (47%)	3.0	0.8	12.0	0.112
**Age category**						
**15–24**	21 (11%)	11 (52%)	1.0			
**25–34**	91 (46%)	36 (40%)	0.5	0.2	1.1	0.067
**35–44**	70 (35%)	32 (46%)	0.4	0.1	1.6	0.217
**45+**	17 (9%)	7 (41%)	0.6	0.1	3.3	0.578
**Education level**						
**Primary or none**	28 (14%)	11 (39%)	1.0			
**Secondary or tertiary**	171 (86%)	75 (44%)	1.1	0.3	3.7	0.862
**Employment**						
**Unemployed**	119 (60%)	51 (43%)	1.0			
**Employed**	80 (40%)	35 (44%)	1.2	0.6	2.8	0.613
**Transport cost**						
**No cost (walkers)**	29 (15%)	13 (45%)	1.0			
**Some cost**	163 (82%)	72 (44%)	0.7	0.4	1.5	0.392
**missing**	7 (4%)	1 (14%)				
**Travel time**						
**0–29 mins**	91 (46%)	41 (45%)	1.0			
**> = 30 mins**	101 (51%)	44 (44%)	1.0	0.5	2.0	0.946
**missing**	7 (4%)	1 (14%)				
**Waiting time**						
**0–2 hr**	66 (33%)	28 (42%)	1.0			
**2–4 hrs**	88 (44%)	41 (47%)	1.3	0.7	2.6	0.429
**>4 hrs**	37 (19%)	16 (43%)	0.9	0.3	2.7	0.840
**missing**	8 (4%)	1 (13%)				
**Know HIV+ person**						
**No one**	44 (22%)	15 (34%)	1.0			
**Someone**	152 (76%)	71 (47%)	0.8	0.2	2.6	0.655
**missing**	3 (2%)	0 (0%)				
**Know someone on ART**						
**No one**	72 (36%)	24 (33%)	1.0			
**Someone**	126 (63%)	62 (49%)	1.6	0.6	4.0	0.369
**missing**	1 (1%)	0 (0%)				
**Knew someone who died of HIV**						
**No one**	96 (48%)	36 (38%)	1.0			
**Someone**	102 (51%)	50 (49%)	1.4	0.5	4.2	0.507
**missing**	1 (1%)	0 (0%)				
**Previous TB treatment**						
**No**	137 (69%)	62 (45%)	1.0			
**Yes**	62 (31%)	24 (39%)	0.7	0.3	1.5	0.326
**Years in community**						
**<5 years**	43 (22%)	24 (56%)	1.0			
**> = 5 years**	154 (77%)	62 (40%)	0.9	0.3	2.5	0.847
**missing**	2 (1%)	0 (0%)				

## Discussion

This study described access to and retention in pre-ART and retention in ART care in eight communities in the Western Cape. This was a community-based rather than facility-based study and therefore included those who did not access care. The results reflected that the majority of people on ART (94%) were retained in ART care whilst less than half (43%) of those not on ART, were retained in pre ART care, although no factors were significantly associated with retention in care in the pre-ART group.

High levels of retention in ART care, as were found in this study, have also been reported by studies in other settings [18]–[20].

The low levels of retention in pre-ART care needs to be improved. In order for patients to be retained in care they need to have access to primary health care facilities, with good quality continuity of care from HIV diagnosis until ART eligibility and initiation. In this study general access to primary health care facilities was demonstrated to be good, with 78 (69%) of those who did not have a CD4 count in the last 6 months having attended a health care facility for any medical reason within the last three months. However the quality of pre-ART care that they received was poor – as they remained without a recent CD4 count. It is essential that pre-ART patients have regular CD4 counts and that ART is initiated as soon as indicated to decrease morbidity and mortality associated with low CD4 counts [3], [5], [21]. Other studies confirm poor retention in and quality of pre-ART care as patients still initiate ART at low baseline CD4 counts (below 200 cells/mm^3^) [5].

In South Africa there is a large gap between the resources that have been expended on the ART programme, as opposed to a pre-ART programme. A comprehensive pre-ART care package that provides quality care to the patient from HCT through to initiation on ART is lacking [9], [22]. Patients are accessing health care facilities, but need to be identified, retained and provided with appropriate pre-ART care. In Cape Town, the policies for pre-ART care are defined but have been incompletely translated into practice [9]. In a 2010 audit on HCT and pre-ART care Scott et al reported many missed opportunities for positive prevention (Family planning, STI screening, PAP smears, and Tuberculosis screening), as well as breaks in continuity of pre-ART care (clinical staging, CD4 count measuring, referral for ART). They report that in Cape Town pre-ART care, in contrast with ART care, has not been allocated additional dedicated health care providers, but has had to rely on existing staff who have been furnished with little scale up support [9]. This is one reason for failure to transform a pre-ART policy into pre-ART care practice.

It is important to determine factors associated with retention in pre-ART care so that patient and provider related factors can be strengthened. In this study, although no statistically significant factors associated with retention in pre-ART care were found, the odds ratios suggested that females and those knowing someone on ART were more likely to be retained in pre-ART care. Likewise, a range of factors associated with retention in or loss to pre-ART care have been described in other studies [6], [11], [12], [23], [24]. In a study in KwaZulu -Natal retention in pre-ART care was associated with female sex as well as a lower initial CD4 count and older age [12]. Factors influencing loss to pre-ART care included distance from the health centre [6], [11] male sex [6], [11], [23] a low CD4 count [6], [11] a history of being treated for TB, referral for HIV testing by a health care provider as opposed to self-referral, weight below 50 kg, unemployment [11] and younger age [23]. A qualitative study, as part of the researching equity in access to healthcare (REACH) project, found that continuing adherence to ART was positively affected by social and economic support by families, friends and the broader community, and negatively by transport and food costs [25]. On-going targeting of vulnerable groups such as men and the youth with initiatives like youth clinics; non medical sites in the community offering HCT and CD4 testing; HIV education and awareness campaigns, and support groups may contribute to improved access and retention in care.

There are several strengths to this study. This was a community-based rather than health facility-based survey and thus participants who had not attended health facilities were also included. To our knowledge no other study determining retention in pre-ART and ART care has sampled in this manner. The communities from which we sampled had been exposed to the ZAMSTAR interventions, and in general participants were knowledgeable about their HIV testing history and were able to provide the researchers with an accurate and comprehensive account of their HIV testing pathway.

There are limitations to this study. The sample size for this study was calculated to answer a different research question and therefore the sample size of the subgroup reported in this analysis might be too small to detect associations with sufficient precision. Data such as CD4 results were self reported and not validated against clinical records. As this was a self reported study factors that may have been associated with retention in care such as initial presenting CD4 count, weight at presentation, and the referral method for HIV testing were unable to be determined from clinical records. There may have been recall as well as reporting bias. This study may not be generalizable to other areas in South Africa as participants may have been more likely to be retained in pre-ART and ART care due to their increased awareness of TB and HIV as a result of their exposure to the ZAMSTAR study interventions and we may have over-estimated retention in care. A further limitation was the low response rate. 66% of the randomly selected adults who were revisited reconfirmed their HIV status and were enrolled. The adults who did not reconfirm their status may have been reluctant to do so because they were not retained in pre-ART or ART care, in which case our study may have over-estimated retention in pre-ART and ART care.

## Conclusion

With the introduction of guidelines enabling all PLWH to access ART with a CD4 count <350 cells/mm^3^ the South African ART programme will continue to expand, and just as retention in ART care is essential to achieve this goal, so too is retention in pre-ART care. This study highlights the need for strengthening pre-ART care. A systematic approach with attention to client and service factors, such as clear pre-ART care policies, staff training and the optimal provision and use of additional resources is needed to increase retention in pre-ART care. This study, shows that missed opportunities will be avoided and clients will be retained in pre-ART care, if care is better integrated, and clinicians attend to the comprehensive needs of clients in primary health care facilities. Further studies, including those of a qualitative nature, that explore reasons for default or non retention in pre-ART care will also give valuable insight as to where to focus resources.
